# "The Hospital's" Special Hospital Sunday Supplement. 1894

**Published:** 1894-06-09

**Authors:** 


### "The Hospital's" Hospital Sunday Fund Supplement, 1894.

### THE FUTURE OF VOLUNTARY HOSPITALS.

### Ex Nihilo Nihil Fit.

That medical voluntaryism should have reached the proportions it has in London is a standing wonder; that it should still outwardly flourish and even appear to be gaining ground is little short of miraculous. But our generation has fallen upon a period of threatened disestablishments and of some actual dissolutions. Much of the talk which alarms the lovers of ordered progress is no doubt mere empty vapouring. But can we be sure that it is all of this character? The certainty seems to be that we can be sure of nothing, at any rate for the next two or three decades. That medical voluntaryism may suddenly collapse is a very manifest possibility. It has hitherto moved forward almost entirely on the two legs of aristocratic superabundance and middle-class generosity. Aristocratic superabundance, with a few conspicuous exceptions, is now unknown. Instead of it we see aristocratic impecuniosity bearing its burdens on every side. Of middle-class generosity the best we can say is that it is fast becoming impossible. *Ex nihilo nihil fit*: a man who has nothing cannot be expected to give. The middle classes are being impoverished by the too often unreasoning demands of the workers; and it need not surprise any student of human nature if one of the first results of this impoverishment should be a determined unconcern for the sufferings and sorrows of the workers.

### Is a Catastrophe Impending?

If it be true that the foundations of medical voluntaryism consist mainly of aristocratic superabundance and middle-class generosity, and that one of these foundations is rusting rapidly away, whilst the other is vibrating dangerously under the repeated heavy blows of trade strikes and hungry industrial demands, it requires no philosopher to perceive that the vast structure, however imposing may be its present proportions and its past history, is in imminent danger of falling. But if this catastrophe be imminent, nay, if it be so much as possible, it is obvious that very serious interests are very fatally threatened. What are those interests, and who are the persons by whom they are represented?

### Threatened Interests.

The collapse of medical voluntaryism would seriously affect at least four classes of persons and interests; and the following seems to us to be the order of their relative importance, from the point of view of public policy: (*a*) Male and female workers, and those who are dependent upon them; (*b*) Hospital officials who earn their bread by the services they render to hospitals; (*c*) The medical men and students who increase their experience or learn the practical details of their art at hospitals; (*d*) Those benevolent persons whose minds dispose them to the maintenance of hospitals as a wise method of charity and duty. Well, but if this be so, then those four classes of persons, at least, should rouse themselves to a full comprehension of the dangers of the situation. Two circumstances in this connection, sound notes of warning. One is that there was a marked falling off in the receipts from the Hospital Sunday collection last year; the other is, that money-raising efforts have been put forth by most hospitals during the past year, which have exceeded in strenuousness and persistency any efforts ever put forth before, but which have been rewarded by poorer results. These are indications which the dullest may comprehend: there are many others which are as plain to the observant eye as the springtime which the swallows have so recently announced,

### Dare We Run the Risk?

But it seems to us that over and above the large numbers of persons whose self-interest must compel them to look with anxiety upon any facts which threaten the stability of voluntary hospitals, the public as a whole has a deep and practical interest in those institutions of the very first order of importance. The work done by hospitals is so immense in volume as to be nothing less than prodigious, and so important in kind as to have hardly any parallel. If this vast and indescribably important work should suddenly cease to be done, or even be greatly limited, the consequences would be such as one absolutely fears to face even in imagination. If the kind of work done by hospitals be considered, how is it possible for its value to the public to be exaggerated? What does it consist of when summarised in a sentence? Not the relief of pain merely, though that includes a whole universe of blessed humanity within itself; but in the saving of limbs, in the carrying of thousands of almost dying persons safely through the fires of deadly fever, in the curing of chronic diseases which kill when they are not cured, in the restoration of sight to innumerable blind, in the salvation of multitudes of worse than homeless women during the agonies of childbirth, in the caring for with soothing tenderness, and the bringing back to life and health with infinitely patient ministrations, thousands of maimed, fever-stricken, congenitally deformed, and otherwise helpless and suffering children. The voluntary hospitals do all this work every day. The fact that they do it, and that they do it every day, is to the thoughtful mind one of the consolations of civilization. The world may be, and often is, a very stern place for the well, but to the sick, in London and throughout Great Britain, it becomes in a moment tender as a mother with her first babe. Is not this something? We repeat that, to the thoughtful mind, it is one of the most refreshing and encouraging of all the achievements of a great and relatively victorious civilization.

### Immeasurably Great.

The nature of the work carried on day by day by hospitals is to the medical mind their most significant feature; but to those innumerable persons who do not and cannot understand the manifold problems of physiology and of the medical art, the vast extent of the work often makes stronger appeal. The barest statement of figures gives in striking outline a body of facts and work the full significance of which the metropolitan mind has never yet so much as attempted to measure. From small beginnings our hospital system has grown so gradually and undemonstratively, that though it is recognized by foreigners as one of the most wonderful features of the modern world, we ourselves not only do not wonder at it, but the majority of us are surprised when we are told that there is anything wonderful about it. But let us make some attempt to realise the meaning and measure of the following figures and facts. Of separate and distinct hospitals and dispensaries in London alone there are 175 (see Burdett's "Hospitals and Charities Annual," p. cclxxxiii.). Practically all of these consist of separate establishments, each with its separate staff of paid officials and its absolutely uncontrolled and independent governing body. With less than half-a-dozen exceptions the whole 175 are supported by voluntary contributions, and the contributions are brought in in almost every case by more or less urgent appeals to the charitable, in other words, by begging. One hundred and seventy-five separate begging establishments, representing in round numbers between one and two millions of individuals of various classes, in London alone. The mere fact is stupendous, whatever view be taken of it. Some will ask why there are so many separate and distinct establishments, and whether a vast number of amalgamations might not diminish cost and increase efficiency. Undoubtedly such critics are right, and we urge upon every lover of medical charities the bending of his mind to the solution of this part of the problem. It is certain that nobody regrets more the unnecessary multiplication of separate hospital establishments than the governors and conductors of the best hospitals.

### General, Special, Miscellaneous.

Of the 175 hospitals and dispensaries in London 32 are more or less "general" in their scope. These general hospitals command the approval of the sound political economist in the highest degree, because they concentrate departments and thereby increase efficiency and diminish cost. The special hospitals number 52. Some of these by their past history and present usefulness have gained both the right and the power to survive. But of many of them it may be said that they are little better than excrescences; and the giving public may congratulate itself that there is one central fund, the Hospital Sunday Fund, which turns a keen and critical eye upon them and their doings. A goodly number of these special hospitals would do well to set their houses in order. There is a day of reckoning approaching for them, or we are very much mistaken. The remaining medical charities consist of 90 dispensaries: institutions these of every conceivable degree of excellence or the want of it. The main point about many of them is that very few persons, that is, responsible persons, know anything whatever about them. But they all beg; we were going to say they were all licensed to beg. But that would have been a mistake. They all beg without licence, the more is the pity; and not only without licence, but without limitation. However much they receive they never have enough.

### Ninety Thousand Modern Miracles.

When we turn from the question of the numbers of these hospitals and dispensaries to their work, the merely critical spirit gives place to another mood. All the hospitals and all the dispensaries do good work, and good work which, for the most part could, under the present system of things, hardly be done elsewhere. The sharpest critic of medical charities cannot deny the excellence of their work, much as he may wish they would concentrate their efforts and manage their affairs according to approved constitutional rules. London alone has in readiness all the year round no fewer than 9,019 beds for in-patients. As many as 90,369 separate individuals availed themselves of those beds in the year 1893. Nearly 2,000 persons in a condition of dangerous or deadly illness were carried to those beds every week, and there each of them received five or six weeks of the best medical treatment and nursing which the world has as yet attained to. Now, practically all this work done for the 90,369 in-patients was not only work of the highest scientific and humanitarian value, but it was indispensable. It was all *bonâ fide* charitable work which commands the approval of all "who know," and which is a glory to British humanity. All the 90,369 were attacked by dangerous, and many of them by deadly illness; and it may be said with the most literal truth that many thousands of them were saved from impending death solely by the promptitude and excellence of the hospital treatment which a charitable public had so opportunely placed at their disposal. This number, 90,000 odd, represents a vast army of sufferers who every year are received by the London hospitals, and, as to the greater number of them, every year are returned to their work cured and well. Of the in-patient work of the London hospitals it is impossible to say too much in praise. It is excellent and indispensable from beginning to end.

### A Department which Might be Better.

The out-patient department of hospitals is at the present moment, and has been for some time, a thorny question; and because it is a thorny question it is desirable that the true and proper constituents of hospitals, the subscribers, should make themselves thoroughly acquainted with it in all its details. It is one of those works of benevolence which, good entirely at the outset, has become by the mere force of a policy of "drift," at best a mixture of good and evil; and in the eyes of many, who are of very competent judgment, the evil is beginning to predominate. It may be thought that it is hardly the way to induce people to give money, to tell them that some fraction of the money may be spent on objects which are not entirely worthy. But, as a matter of duty and policy, we feel that the right course at a crisis like the present is to take the public into full confidence, and to ask it to use all its best and most intelligent efforts in the solution of a very difficult problem. We shall give the figures for the out-patient department. They will not be given for the purpose of making a puffing display of hospital work; but because they represent the actual facts. In our opinion they are too large; and, as we have said, we hope the constituents of hospitals will help to devise methods for their diminution. The total number of out-patients who attended at the various London hospitals during 1893 was 1,562,066 (one million five hundred and sixty-two thousand and sixty-six). But the whole population of the metropolis only amounted to 4,211,056. So that not less than 399 persons in every thousand of the population of London were recipients of medical charity last year; rather more than one in every three. For the moment we leave these figures here to speak for themselves. We merely suggest that it is not a comfortable fact, either for the political economist or the hospital subscriber of the future, that one in every three of the inhabitants of the metropolis should be willing to accept medical charity.

### Priced and Priceless.

The money value of the medical relief given annually in London can never be made known, because the most expensive part of it, which consists in the skilled services of the physicians and surgeons of the hospitals, costs neither the charitable public nor the patients anything at all: it is a free gift made by the physicians and surgeons. Of course, it may be said that those gentlemen have their *quid pro quo*; and that we are not concerned to deny. But that is not the question which is now under consideration. For practical purposes the charitable public and the working classes command the best services of the most skilful and scientific physicians and surgeons in the world free of cost. The actual expenditure of the hospitals for maintenance, drugs, instruments, nursing, and ten thousand other necessaries can easily be arrived at, and is published, here and elsewhere, every year. The returns for 1893 show that the various general and special hospitals, sick institutions and dispensaries of London, spent for maintenance the vast sum of £689,863. Of this sum the various districts spent in the following measure: Newington and South District, £92,867; City and East Central, £55,674; Westminster, £89,353; St. Marylebone and West Central, £113,599; Kensington and West, £147,908; Islington and North West, £75,253; Stratford and East End, £115,209.

### The "Seven Circles."

These three sets of facts, dealing with the in-patients, the out-patients, and the annual expenditure of the hospitals, give at least an outline of the whole hospital world and its activity. They do not and cannot give more than an outline. The details, which are innumerable and full of the profoundest human interest, only reveal themselves fully to the careful student who spends a lifetime within hospital walls. But we may be helped to form a little more definite idea of the vast and detailed work which the hospitals are doing if we remember that all this charitable activity is carried on, and all this money is spent, within a radius of eight or nine miles from Charing Cross. If we should take a point near Charing Cross as the centre of London, and draw seven concentric circles round it, the first circle having a diameter of two miles, and the remaining circles increasing each in diameter by two miles, so that the circumferenee of no circle is more than a mile distant from that of any other circle, and that of the inner circle not more than a mile from the centre at Charing Cross, we could, by a mere redistribution of existing hospital accommodation, place 300 hospitals, containing over thirty beds each, in these various circles, so that no one hospital should be more than a mile distant from another, and no inhabitant of Greater London should be more than half a mile distant from a fully-equipped hospital of more than thirty beds. At the present time there are beds enough in the existing London hospitals to effect all this, and a charitable revenue almost large enough to support them all. And this in-patient work, which is represented by hospital beds, be it remembered, has the entire approval of all classes of the community, even of those who set no bounds to their condemnation of the out-patient system.

### Our Fathers Built: Shall We Destroy?

We have been at some pains to make non-medical readers understand to some extent the magnitude, as well as the nature, of the work which is carried out by the London hospitals at the cost of the charitable, and for the advantage of the whole community. Our object is to enable the public to see clearly what it is which is endangered when we speak of the possible collapse of the hospital system. Here is a system, not of scientific and benevolent work merely, but of work which is done at great cost, and the resources for which have only been built up by very slow degrees, and with infinite pains, by many generations. The two-fold necessity of the moment is then, not the doing of the work alone, but the preservation of the resources which pay for the work, and the insurance of their continuance. We repeat that there are not wanting indications of collapse in the resources which maintain the work of hospitals. The public, we are certain, do not desire the collapse of hospital resources, or even the diminution of hospital work. There is then this problem for solution: What are the future prospects of voluntary hospitals; and how may the resources of those hospitals be rendered adequate and permanently secure for the certainly increasing necessities of the present and the immediate future?

### Unification.

The only solution of this problem is to reconstruct and unify the hospital system on such politico-economic principles as will approve themselves, first of all to the medical profession, and secondly, to all the thinking portion of the community. The hospital system is what it is by its own spontaneous growth, and by a process of severe political neglect. This is the explanation of many of its imperfections. It cannot sustain this neglect any longer. It is too large, and demands too large resources for its maintenance. A generation of prosperous men and women may give money for institutions which are small and make small demands. But when institutions become enormously large, and make correspondingly enormous demands for their maintenance; when, too, a generation has ceased to be prosperous, and has become straitened in every dimension of its giving capacity, then it pulls itself together and demands to know—why? when importunity pleads for enlarging doles of wealth. In our judgment the community not only will not, it cannot continue for ever to support our hospital system on its present huge, floundering, unconcentrated, and costly basis.

### Constitutionalism.

Hospital authorities must take their business firmly in hand and set it in order. If they do not, the diminishing springs of wealth will dry up for them altogether, and their institutions will be left stranded and mere ghosts of their former selves. There are many details of administration which require to be amended, chief among which are the disestablishment of superfluous special hospitals, the improvement of general hospitals by the addition of special departments, the establishment of fully-equipped hospitals at more numerous and more convenient centres, the control of unlicensed begging, and so on. But the one great want of the hospital system of London is central government. The present system of hospital management is a system of decentralisation which is rushing down to chaos, a system of "Home Rule" run mad.

### To Fair-minded Englishmen.

In pleading in this candid way for all the sympathy and help which a generous, if not very prosperous, public can give to the Hospital Sunday collections, we have made our appeal to the fair-mindedness and common sense of the English people. We have affirmed that the work of the hospitals is in all respects great and noble, and that the hospital system, though not perfect, is fairly efficient in practice, but may be made to work much better. The special claim of the Hospital Sunday Fund at this moment is, not merely that it raises much-needed money for the hospitals, but that it aims in a quiet, sober, and sympathetic spirit to control vagaries, to introduce reforms, and to bring about precisely that condition of central administration on sound constitutional principles which is here advocated. A liberal Hospital Sunday collection strengthens the Hospital Sunday Fund; and the strengthening of that fund is the strengthening of public control over hospital affairs and administration. The two things of all others, we emphasise, which hospitals need at the present moment are increased public interest in their work and government, and increased support. Increased public interest in their work and government is the more urgent necessity of the two. Hospitals belong to the public, they are a noble public possession, they improve the public character, they gladden the public heart. It is for the public, and the whole public, to claim its share in their work and their government. When this is done with the thoroughness and determination which the Englishman puts into all the business which he seriously takes in hand, there will not fail the golden showers of indispensable material support.

### SUMMARY OF WORK DONE DURING 1893.

It will be seen from the following summary that the Voluntary Hospitals and Medical Charities of London, during the twelve months ending December 31st, 1893, relieved over one million three hundred and ninety-two thousand patients at a cost of £689,863. The Ordinary Income only amounted to £516,375, leaving a deficiency of £173,490 on the year's work. The Legacies received in 1893 amounted to £150,803, being £126,000 less than in 1892, **thus leaving a net deficiency in the year's working of Twenty-two Thousand Six Hundred and Eighty-two Pounds.**

| No. of Beds. | No. of Beds Daily Occupied. | Hospitals and Dispensaries. | In-patients. | Out-patients. | Total Expenditure. | Income. | | | Total Income. | Legacies not included in previous column. |
|---|---|---|---|---|---|---|---|---|---|---|
| | | | | | | Charitable. | Proprietary. | Patients' Payments. | | |
| | | | | | £ | £ | £ | £ | £ | £ |
| 1,503 | 1,100 | Newington and South District ... | 14,893 | 201,513 | 92,867 | 36,163 | 37,794 | 11,665 | 85,622 | 1,207 |
| 534 | 396 | City and East Central District ... | 6,541 | 153,403 | 55,674 | 27,276 | 8,009 | 4,776 | 40,061 | 28,537 |
| 993 | 826 | Westminster District ... ... | 11,313 | 157,932 | 89,353 | 38,098 | 13,055 | 11,833 | 62,986 | 14,027 |
| 1,378 | 1,044 | St. Marylebone and West Central District ... ... ... ... | 10,240 | 186,244 | 113,599 | 43,951 | 20,407 | 9,048 | 73,406 | 16,192 |
| 1,636 | 1,411 | Kensington and West District ... | 16,198 | 209,129 | 147,908 | 66,506 | 32,215 | 9,370 | 108,091 | 34,475 |
| 835 | 628 | Islington and North-West District | 8,491 | 149,755 | 75,253 | 34,369 | 10,974 | 7,404 | 52 747 | 13,167 |
| 1,333 | 1,067 | Stratford and East-End District... | 15,665 | 250,837 | 115,209 | 64,142 | 27,941 | 1,379 | 93,462 | 43,197 |
| 8,212 | 6,472 | | 83,341 | 1,308,813 | 689,863 | 310,505 | 150,395 | 55,475 | 516,375 | 150,802 |

### THE LORD MAYOR'S APPEAL.

Sir,—We are once more on the eve of Hospital Sunday in London, and for the twenty-second year in succession the courtesy of your assistance is asked to enable me in my capacity as treasurer of the Fund to make a very urgent appeal to the charitable public to support this annual effort in aid of the hospitals, dispensaries, and convalescent institutions, which are so continuously and so largely providing for the wants of the sick poor of the metropolis.

Last year the Fund reached a total of £39,290, being less than has been raised in any year since 1885, and this amount was distributed amongst 122 hospitals and 55 dispepsaries. It is computed that at least £100,000 is needed to clear these institutions from debts incurred beyond their normal incomes in the medical and surgical treatment of the London poor. An interesting return recently published in the *Lancet* shows that last year no fewer than 103,585 in-patients and 3,871,290 out-patients were treated in the various hospitals, in addition to 243,801 accident and emergency cases. In spite of this it is a fact that whereas 200 years ago the hospitals in London could take in one sick person out of every 133 residents, the proportion is now one out of every 660. But the good done by the hospitals cannot be estimated by mere statistics of treatment, for, as the same article points out—"New principles in medicine, a new era in surgery, and a clearer comprehension of the laws of health and of the secret processes of disease are the offspring of the tenderness, skill, and assiduity with which the sick poor has been cared for in the hospitals. Sanitary laws have been formulated, and although even now these are often very inadequately applied, their absence, if hospitals were abolished in London altogether, would reduce us from the present high degree of civilisation, for which we are in great part indebted to the hospitals, again to a condition little short of barbarism."

For these reasons I venture, with your permission, to prefer an especially pressing appeal for the benevolence of the various congregations on Hospital Sunday. It is the one opportunity given in each year for the churches of the metropolis through their ministers and laity to join in an expression of gratitude for the good work effected by the London hospitals, and also of thankfulness for the immunity from sickness and accident which in our own families we have enjoyed.

I would, therefore, very earnestly plead for a hearty and munificent response to the appeals which will be made from the pulpit on Hospital Sunday, and I would add the usual intimation that I shall be glad to receive at the Mansion House any donations towards the fund which may be sent to me.—I am, sir, your obedient servant,

George Robert Tyler, Lord Mayor.

The Mansion House, June 7th, 1894.

